# Using experimental de-worming to measure the immunological and pathological impacts of lungworm infection in cane toads

**DOI:** 10.1016/j.ijppaw.2017.09.006

**Published:** 2017-09-20

**Authors:** Patrick B. Finnerty, Catherine M. Shilton, Richard Shine, Gregory P. Brown

**Affiliations:** a School of Life and Environmental Sciences, University of Sydney, Sydney, New South Wales 2006, Australia; b Berrimah Veterinary Laboratories, Northern Territory Government, Berrimah, Northern Territory, 0828, Australia

**Keywords:** Rhinella marina, *Bufo marinus*, Host, Parasite, Nematode

## Abstract

The immunological and pathological consequences of parasite infection can be more rigorously assessed from experimental manipulation than from correlational studies of natural infections. We used anthelmintic treatment to experimentally decrease intensities of lungworm infection in captive and free-ranging wild cane toads to assess parasite impacts on host immune responses. First, we administered the anthelmintic drug Ivermectin to both infected and uninfected toads, to distinguish drug effects *per se* from the impacts of killing lungworms. Worms began dying and decomposing <48 h after injection. The only immunological variables that were affected by anthelmintic treatment were bactericidal capacity of the blood which increased in parasitized toads (presumably triggered by decomposing worms in the lungs), and the phagocytic capacity of blood (which increased in both infected and uninfected toads); the latter effect presumably was caused by the injection of Ivermectin *per se* rather than removal of parasites. Second, we looked at correlates of variation in the infection intensity induced by de-worming (in both captive and free-ranging toads) over an eight-week period. Heavier lungworm infection was associated with increased phagocytic ability of the host's blood, and a reduction in the host's liver mass (and hence, energy stores). Experimental de-worming thus revealed pathological and immunological costs of the presence of lungworms, and of their removal by anthelmintic injection.

## Highlights

• Minor short-term immune responses to anthelmintics and lungworm decomposition.
• Long-term lungworm infection saw minor pathological impacts on lung/colon tissue.
• Heavier infection intensity saw increased whole blood phagocytic ability.
• Heavier infection intensity associated with reduction in liver mass (energy stores).
• De-worming revealed pathological costs of infection and anthelmintic treatment.

## Graphical abstract

**Figure:**
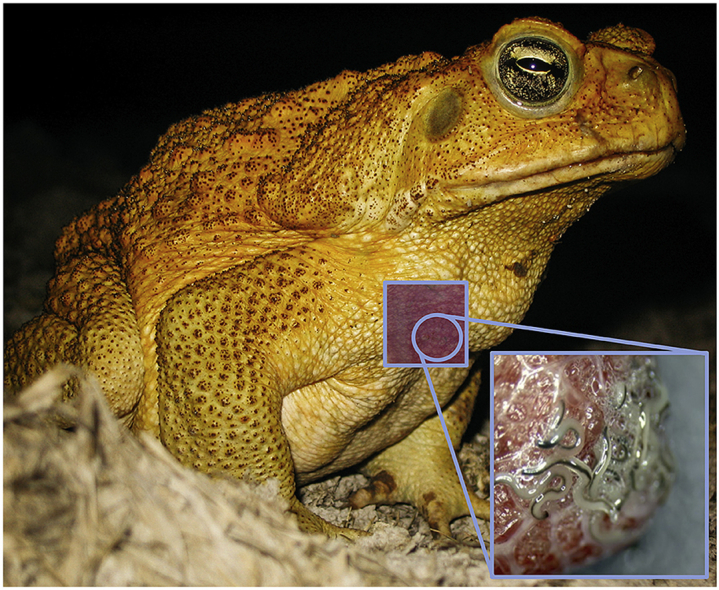

## 1 Introduction

Host–parasite biology has been studied for many years, but the ecological impacts of parasites on their hosts have become a major focus only recently (Thompson et al., 2010, Gómez and Nichols, 2013, Jenkins et al., 2015, Polley and Thompson, 2015). Correlations between parasite infections and host biology, although relatively straightforward to document, provide only a weak basis for inferences about causation (Brown et al., 2015a). For example, animals with heavy parasite infections may also be in a weakened condition. But from this observation it is not possible to ascertain if the parasites cause the weakness, or if some other factor causes weakness and the weakened animals become more prone to parasite infections. A more powerful method is to experimentally manipulate infection status and monitor the results, ideally in free-ranging hosts in order to document effects under ecologically relevant conditions (Kelehear et al., 2011, Heise-Pavlov et al., 2014).

One means of experimentally manipulating parasite levels is by exposing naïve hosts to infective larvae. This method is ideal for studying responses to the initial establishment stages of infection (Pizzatto et al., 2010, Kelehear et al., 2011, Nelson et al., 2015) but may be less feasible in studying longer-term effects of chronic infection, especially if the parasite has an extended lifespan. Experimentally eradicating parasites from hosts using parasite-specific drugs developed for use in domestic animals (Stien et al., 2002, Pedersen and Antonovics, 2013) is an alternative approach that selectively removes adult parasites from a subset of infected hosts. This experimental removal of parasites may also overcome ethical and logistical difficulties associated with deliberately infecting animals. Experimental de-worming has been used with great success in several wildlife studies. For example, de-worming increased the time spent moving and foraging by wild Grant's gazelles, *Nanger granti* (Worsley-Tonks and Ezenwa, 2015), increased host body mass and fecundity in wild reindeer, *Rangifer tarandus* (Stien et al., 2002), enhanced breeding success in free-ranging pheasants, *Phasianus colchicus* (Draycott et al., 2006), increased survival in free-ranging African buffalo (Ezenwa and Jolles, 2015), and altered the parasitic community in free-ranging whited footed and deer mice (Pedersen and Antonovics, 2013).

If we are to use anthelmintic drugs to quantify the impacts of parasitism on host behavior and performance, we need to understand the consequences of those drugs on host physiology. For example, are impacts of de-worming on the host mediated by shifts in immune-system functioning, and/or by the inflammation induced by decomposing parasites within the host's body? Our current understanding of parasite–host interactions is focused on the parasite and the resultant changes in host behavior, performance, reproductive output, and demographic traits (Bakker et al., 1997, Fenner and Bull, 2008, Kelehear et al., 2011). Understanding the pathological and immunological consequences of parasitism on a host can clarify the processes by which parasites induce these specific changes.

As part of a study to quantify the behavioral and ecological effects of removing lungworms (*Rhabdias pseudosphaerocephala*) from cane toads (*Rhinella marina*), we quantified aspects of immune system responses and morphological changes associated with (i) injection of the anthelmintic drug Ivermectin, (ii) decomposition of parasites in the host's lungs, and (iii) long-term variation in parasitic infection intensity. We assessed the effects of de-worming over two time periods:

(1) Short-term (<1-week) effects of experimental de-worming on infected versus non-infected toads, to separate effects of the anthelmintic drug itself versus the effect of the drug plus decomposing worms on host immunological responses (concentrations of blood cells and bactericidal ability), and the pathological effects of *R. pseudosphaerocephala* infection on lung tissue (Kucik et al., 2004, Turner et al., 2010);
(2) Long-term (>2-month) effects of experimental de-worming (which generated variation in parasitic infection intensity among hosts) on organ mass, colonic tissues (the site of larvae shed by those adult worms), and immune responses (concentrations of blood cells and bactericidal ability) of free-ranging and captive cane toads. Do histological, physiological and pathological responses differ between toads of varied infection intensities several months after hosts are subjected to ‘de-worming’?

## 2 Materials and methods

### 2.1 Host–parasite system

Cane toads (*Rhinella marina,* formerly *Bufo marinus*) are large (up to 500 g) toxic bufonid anurans native to Central and South America. Since being introduced into Australia in 1935, these toads have caused the decline of many populations of endemic predators that lack physiological resistance to the toad's powerful bufotoxins (Smith and Phillips, 2006, Jolly et al., 2015).

The lung nematode *Rhabdias pseudosphaerocephala* is found through most of the cane toad's Australian range (Dubey and Shine, 2008), but is absent from the expanding invasion front (Phillips et al., 2010). *Rhabdias* nematodes have a direct life cycle (Baker, 1979). Briefly, hermaphroditic adults inside the toad's lungs lay eggs that pass into the toad's digestive tract and hatch into first-stage male and female forms which are free-living in the soil once they are defecated by the toad. These larvae mate to produce infective third-stage larvae (L3) which develop inside free-living females. After 3–4 days the larvae break free from the mother and are released into the soil (Baker, 1979). When an L3 locates an anuran host it pierces through the skin, alimentary tract or membrane behind the eye and burrows through tissue to reach the lungs of the toad where it feeds on blood (Pizzatto et al., 2010). After they reach the host's lungs the parasites mature and begin producing eggs in as little as 5 days (Kelehear et al., 2012). Although infection dynamics can vary climatically and seasonally (Barton, 1998, Pizzatto et al., 2013), up to 80% of cane toads are infected in populations in far north Queensland (Barton, 1998), with infection intensity reaching up to 282 adult worms per host (Pizzatto et al., 2013).

### 2.2 Study site

Our study took place between August and December 2016 at Leaning Tree Lagoon (12.7 °S, 131.4 °W) and nearby areas in the Adelaide River floodplain, Northern Territory, Australia. Leaning Tree Lagoon is a 6-ha billabong situated 80 km south-east of Darwin. The area experiences a tropical climate that is dry for about half of the year and wet for the other half, with monsoonal rainfall between November and April (Shine and Brown, 2008). Our study took place primarily over the dry-season (May–November). Average maximum temperature surpassed 35 °C each month and the mean monthly minimum temperature between August and November was 21 °C (BOM, 2016). Cane toads appeared in the area late in 2005, and lungworms were first recorded in toads in the area in 2008 (Phillips et al., 2010).

### 2.3 Short-term effects of de-worming on immune responses of adult toads

We collected 16 toads from Leaning Tree Lagoon on the night of 5th December 2016. Toads were housed individually in 300 × 200 × 200 mm plastic boxes and fed four large adult crickets daily. We examined at least three fecal samples (obtained on different days) per individual for the presence of *R. pseudosphaerocephala* larvae to assay their infection status. The identification of *Rhabdias* (L3) larvae as *R. pseudosphaerocephala* was confirmed under a dissecting microscope based on their shape, size and movement patterns. No other known cane toad parasites in Australia resemble *R. pseudosphaerocephala* larvae, or have been recorded in cane toad feces. At initial capture, 10 of the 16 animals were infected with *Rhabdias* and six were not. We injected toads with Ivermectin (0.02 mg/100 g toad; Ivomec^©^, Merial, Duluth, USA) over a range of times prior to euthanasia. Five toads (3 infected, 2 non-infected) were injected 7 days prior to euthanasia, four toads (3 infected, 1 non-infected) 4 days prior to euthanasia, three toads (2 infected, 1 non-infected) 2 days prior to euthanasia, two toads (1 infected, 1 non-infected) 1 day prior to euthanasia, and two toads (1 infected, 1 non-infected) were euthanized but not injected with de-wormer. Thus, at the time of euthanasia, toads had been treated with Ivermectin 1, 2, 4 or 7 days previously, and two toads had not been treated at all. Blood samples were immediately collected from euthanized toads for use in immune assays (see below).

### 2.4 Histological analysis of the short-term effects of de-worming adult toads

Lungs of the 16 captive toads were removed at the main stem bronchus and injected with 10% phosphate-buffered formalin prior to being placed in a jar of the same fixative. After fixation, lungs were incised lengthwise and worms floating free in the lungs were removed, counted and retained. For histological processing, one lung from each toad was sectioned transversely four times, at equal distances along its length. These four transverse sections were processed in a standard fashion (see below) for histological examination, and 5 μm sections stained with hematoxylin and eosin were cut, resulting in one slide for each of the four transverse sections from one toad. Lung slides were examined by a pathologist (C.S.) who was blind to the treatment group of the toad. Lung sections on each slide were screened at 100× magnification and areas of inflammation were categorized based on the predominant cell types involved in those inflammatory reactions. Two types of areas of inflammation were identified:

(i) lymphocytic inflammation – focal interstitial aggregates of 10–20 small mononuclear cells; mostly lymphocytes, with a few admixed macrophages.
(ii) neutrophilic inflammation – focal intraluminal loose aggregates of 5–20 neutrophils, often mixed with red blood cells and macrophages (Santos et al., 2016).

For histological processing, the tiny size of the nematodes necessitated embedding them in agar before cutting slides. Larvae were fixed in 10% buffered formalin for 24 h and then embedded in agarose agar (0.5 g of agarose mixed with 50 mL hot water) in a small rectangular (2 × 3 cm) shallow (0.5 cm deep) mold. Once set, the agar containing the larvae was removed from the mold using a scalpel and placed in a beaker of methanol for 2 h to harden. The resultant piece of agar containing embedded larvae was then placed in a histology cassette, processed in standard fashion (see below) for histology, embedded in paraffin, sectioned and stained. *Rhabdias* were examined, blind to the toad treatment group, and viability scored as follows: 0 = non-viable (indistinct staining, generally pale, eosinophilic, cells with indistinct boundaries, poorly delineated nuclei, blurred cuticular layers, and hypodermal boundaries); 1 = intermediate (transitional between features from categories 0 and 2); and 2 = viable (cells were well-delineated and well-stained, with distinct nuclei, cuticle layers distinct, with a sharp boundary between the hypodermal cells and underlying coelom).

### 2.5 Long-term effects of de-worming on immune responses of adult toads

#### 2.5.1 Captive cane toads

We captured 49 toads over three nights from multiple sites on the Adelaide River floodplain (12.6 °S, 131.3 °W) in late August 2017. Toads were taken to the laboratory where they were weighed, measured (snout-urostyle length [SUL], head width and right tibia length) and given a unique toe-clip. Toads were individually housed in plastic containers (300 × 200 × 200 mm) lined with newspaper. Water was provided in a shallow earthenware dish and toads were fed four large crickets every second day. We determined whether each toad was infected with *Rhabdias* by inspecting its feces under a dissecting microscope. Uninfected and infected toads were randomly assigned to receive either two doses (once at capture, and again 2 weeks later) of Ivermectin (0.02 mg/100 g toad), or a same-sized control dose of Amphibian Ringer's solution. This resulted in four treatment groups: ID = infected and de-wormed (n = 11 toads), IC = infected and Amphibian Ringer's control (n = 13), ND = non-infected and de-wormed (n = 13), NC = non-infected and Amphibian Ringer's control (n = 12). Over the next four months (Aug–Nov 2016) these captive toads were subjected to trials of physical performance and behavior (Finnerty et al., 2017). After the trial period, all 49 captive toads were euthanized and dissected. Nineteen of these captive toads (n = 8 infected [IC], n = 11 non-infected [ID]) were used in immune assays and all 49 were dissected to compare liver masses (see below).

#### 2.5.2 Free-ranging cane toads

Over a 12-week mark-recapture study at Leaning Tree Lagoon between August and November 2016, we determined the infection status of 454 toads (Finnerty et al., 2017). At initial capture, the infection status of each toad was assessed by inspecting fecal samples after which each toad was randomly assigned to receive either an injection of anthelmintic Ivermectin (0.02 mg/100 g toad) or a control injection of Amphibian Ringer's solution (Wright and Whitaker, 2001). This resulted in the same four treatment groups as above in Section 2.5.1. At recapture, each toad was re-dosed with the same treatment it received at its initial capture. Individual toads were recaptured and re-dosed up to four times. At the end of the 12-week mark-recapture study, six IC (i.e. not de-wormed) toads and five ID (i.e. de-wormed) toads were fitted with radio-transmitters and tracked for 5 days. These 11 toads were then recaptured and taken back to the laboratory, euthanized, blood samples were taken for use in immune assays, and all 11 toads were dissected (see below in Section 2.7). Elsewhere (Finnerty, 2017), we describe the propensity for toads infected with *Rhabdias* to produce feces with a higher water content than that of non-infected toads. To determine whether this could be caused by colonic irritation (possibly in response to the presence of motile *Rhabdias* larvae), we also collected colon samples from these 11 free-ranging toads for histological examination.

### 2.6 Dissection and histological methodology

Post euthanasia and after the removal of blood samples from both captive and free-ranging toads, toads were dissected via a mid-ventral incision and we recorded carcass mass, liver mass (±0.001 g) and infection intensity (number of lungworms in lungs). Livers were dabbed dry on paper towel to remove excess fluid before being weighed. Samples of colonic wall were fixed in formalin, sectioned and stained for histological examination (see below). Sections of the colon were inspected for epithelial differentiation and the presence of necrotic cells. Lymphoplasmacytic and granulocytic infiltrations of the mucosa were scored (1 = mild, 2 = moderate, 3 = marked).

Toad tissues and lungworm larvae were processed for histological examination using standard techniques. To make histology slides, formalin-fixed toad tissues were appropriately trimmed, placed in histology cassettes and processed in standard fashion. Processing involves a series of dehydrating steps accomplished by passing the tissues through increasingly concentrated ethanol solutions, followed by clearing using limonene, and impregnation with paraffin wax. Processed tissues were then embedded in paraffin wax blocks and 5 μm sections cut using a microtome, placed on glass slides, stained with hematoxylin and eosin and a cover slip fixed in place using permanent mounting medium.

### 2.7 Euthanasia, blood sampling and immune assays

We euthanized toads with an overdose of pentobarbital sodium (50% Lethabarb diluted in water: Pizzatto et al., 2013) and then removed 0.3-mL blood samples via cardiac puncture. Each sample was refrigerated immediately and immune assays began as soon as the blood sample was collected from the last toad. Blood samples were used to (1) measure numbers of each leukocyte cell type (i.e. basophil, eosinophil, monocyte, lymphocyte, neutrophil), (2) assay whole blood phagocytic activity, (3) measure concentrations of white and red blood cells, and (4) assay bacteria-killing ability of plasma. This combination of measures reflects the configuration of standing constitutive lines of immune defense (relative leukocyte concentrations) as well as host ability to react to experimentally induced immune challenges (phagocytosis and bacteria-killing assays). Methods are explained in detail elsewhere (Brown and Shine, 2014, Brown et al., 2015b, Brown et al., 2016). Briefly, the methodology used was as follows:

(1) Blood smears were prepared and stained using modified Wright's stain. Slides were examined under 1000× magnification to identify leucocytes, which were classified as either lymphocytes or neutrophils (Brown et al., 2015b).
(2) We measured light emitted from the chemical reactions that occur when blood cells (mainly neutrophils) phagocytize pathogen particles. The pathogen particles used were Zymosan (Sigma Z4250, from yeast cells). We added Luminol (Sigma A8511) to amplify the photons produced by the reaction to detectable levels. Using sterile Amphibian Ringer's solution, whole blood was diluted 1:20 and 240 μL was added to the wells of a 96-well assay plate. 10 μL of a 20-mg/mL solution of Zymosan (in phosphate-buffered saline [PBS]) and 30 μL of a 10-mM solution of Luminol (in 0.2 M sodium borate buffer) was then added to each well. For each sample prepared we added a negative control well by substituting 10 μL PBS in place of Zymosan. The plate was placed in a luminometer (BMG Labtech, Ortenberg, Germany) where light emissions were recorded over 160 min. From the light emission time series we calculated average emission over 160 min, maximum emission, and time of maximum emission (Brown et al., 2015b) for statistical analysis. However, for the short-term study a power failure truncated this assay after only 95 min. It was not possible to determine the time at which the maximum level of luminescence would have occurred for these toads. Therefore, only the mean and maximum levels of luminescence during the 95-min assay period could be calculated for each sample.
(3) Hemocytometry was used to quantify concentrations of red and white cells in toad blood samples. 0.05 mL of whole blood was diluted 1:200 in Natt-Herrick's stain. 0.1 mL of the solution was placed into a hemocytometry chamber and inspected under 400× magnification. Red and white blood cells were counted to estimate cell concentrations (Wright and Whitaker, 2001).
(4) We measured bactericidal capacity of soluble immune products in blood plasma (Brown et al., 2015b). After blood samples were centrifuged for 4 min, we removed the plasma portion and diluted it 1:20 in CO_2_ independent medium. In distilled water, we rehydrated a lyophilized pellet of *Escherichia coli* (ATCC 8739, Microbiologics, USA) so a 10-μL sample contained approximately 600 colony-forming units (CFUs). As an immune ‘challenge’, we added 10 μL of the *E. coli* suspension to 140 μL of each diluted plasma sample. We then spread 50 μL of the plasma–bacteria mixture onto a tryptone soya agar plate. We incubated the remaining plasma–bacteria mixture for 1 h at 25 °C, then spread a 50-μL aliquot onto a second agar plate. Using samples of CO_2_ independent medium (without plasma) after 0 min and 60 min incubating with 600 CFU's of *E. coli,* control plates were prepared in a similar manner. All plates were then incubated for 24 h at 37 °C. The total number of *E. coli* colonies on each plate was counted, and the % change in colonies after 60 min of incubation with plasma was calculated as:

$$\left(\frac{\left(60\min count-0\min count\right)}{\left(0\min count\right)}\right)\times \;100$$

### 2.8 Statistical analyses

#### 2.8.1 Histological analysis of short-term effects of de-worming

Because of small sample sizes and non-normal distribution of data we used non-parametric Spearman's correlations to explore relationships between histological measures and infection intensity.

#### 2.8.2 Immune assay analysis

To assess the short-term effects of Ivermectin injection on immune and bactericidal responses we used initial infection status (infected vs. non-infected), number of days since injection, and their interaction as factors in the analyses. We used linear mixed effects models fit by restricted maximum likelihood estimation (REML) to investigate the main effects of de-worming on immune and bactericidal responses over both the short-term and the long-term.

Mean and maximum luminescence values and counts of *Rhabdias* in the lungs were log-transformed prior to analysis to normalize their distributions. Data from the 19 captive and 11 free-ranging wild toads were pooled into a common analysis to assess the effects of parasite infection on blood cell parameters and immune assay responses. The source of the toads (captive vs. free-ranging) was included as an independent variable in the analysis. We used actual counts of *Rhabdias* infection intensity as a second independent variable in the analysis, along with its interaction with toad source. Thus we used variation in infection intensity caused by anthelmintic treatment rather than ‘treatment’ *per se* as an independent variable in the analyses.

#### 2.8.3 Dissection analysis

To investigate the effect of *R. pseudosphaerocephala* infection on relative liver mass of toads, we used an ANCOVA with ‘infection group’ (de-wormed vs. not de-wormed) as a fixed factor and carcass mass as a covariate. To explore the possibility that relative liver mass was affected by the number of lungworms (rather than simply presence/absence of these parasites), we repeated these analyses with infection intensity as the independent variable. Toads without any lungworms were excluded from this latter analysis.

In all cases, we inspected residuals from analyses for violations of test assumptions. Where necessary, data were log-transformed to better meet assumptions of normality and homogeneity of variance. For all analyses where group had a significant effect in the model, we conducted Tukey HSD post-hoc tests to locate significant differences (indicated by alphabetical superscript in figures). All analyses were performed with JMP Pro 11 (SAS Institute, Cary, NC).

## 3 Results

### 3.1 Short-term effects of de-worming on immune response

**Fig. 1:**
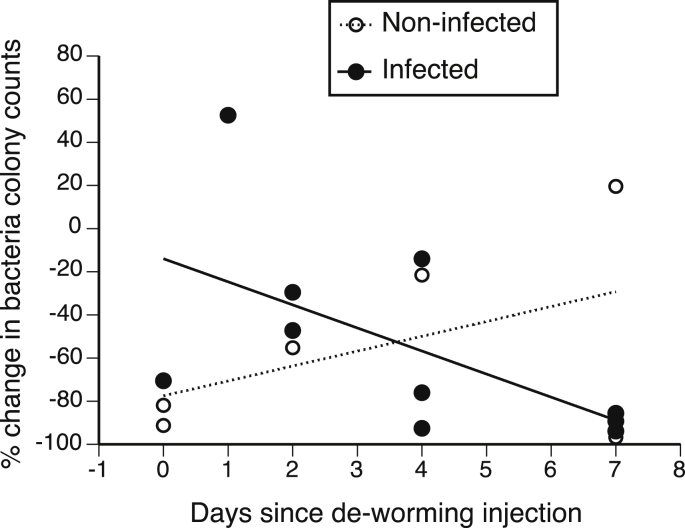
Effects of time since anthelmintic administration on the bactericidal ability of the blood of 16 cane toads. Based on fecal examination, 10 of the toads had lungworm infections at the time of injection and 6 did not.

Infection status at time of treatment (infected vs. uninfected) did not influence blood cell measures in the days following anthelmintic injection (Table 1). However, the maximum level of phagocytosis increased with time since Ivermectin injection (Table 1), in both infected and uninfected toads. Bacteria-killing ability was affected by an interaction between infection status and the time since Ivermectin injection. Bactericidal ability increased with time since injection in infected toads, but decreased in non-infected toads (Table 1, Fig. 1).

**Table 1:** Effects of lungworm infection status at time of anthelmintic treatment (present vs. absent), and time since anthelmintic treatment on concentrations of blood cells and phagocytic and bactericidal ability of the blood in adult cane toads (*Rhinella marina*). Significant values (p < 0.05) are shown in boldface font.

Trait	Effect	F1,12	p
**Blood cell density (per mL)**			
Erythrocytes	Infection status	0.93	0.35
	Days post injection	0.75	0.40
	Infection status × days post injection	0.04	0.85
ln-leukocytes	Infection status	0.01	0.92
	Days post injection	0.17	0.69
	Infection status × days post injection	0.21	0.65
ln-neutrophils	Infection status	1.74	0.21
	Days post injection	0.13	0.72
	Infection status × days post injection	0.10	0.76
ln-lymphocytes	Infection status	1.29	0.28
	Days post injection	0.05	0.83
	Infection status × days post injection	0.00	0.97
Neutrophil:lymphocyte ratio	Infection status	1.67	0.22
	Days post injection	0.03	0.87
	Infection status × days post injection	0.12	0.73
**Immune assay measure**			
log10 mean luminescence	Infection status	1.91	0.19
	Days post injection	3.64	0.08
	Infection status × days post injection	0.07	0.80
log10 max luminescence	Infection status	3.06	0.10
	Days post injection	5.00	**0.04**
	Infection status × days post injection	0.13	0.72
Bactericidal ability	Infection status	0.00	0.99
	Days post injection	0.23	0.64
	Infection status × days post injection	4.90	**0.04**

‘Days post injection’ refers to the number of days (1, 2, 4 or 7 days, see Section 2.3) between the time when toads had been treated with anthelmintic and the time of euthanasia and blood sampling to. Two toads had not been treated with anthelmintic when euthanized and were given scores of 0 days post injection.

### 3.2 Histological analysis of short-term effects of de-worming

#### 3.2.1 Evidence of autolysis of lungworms

**Fig. 2:**
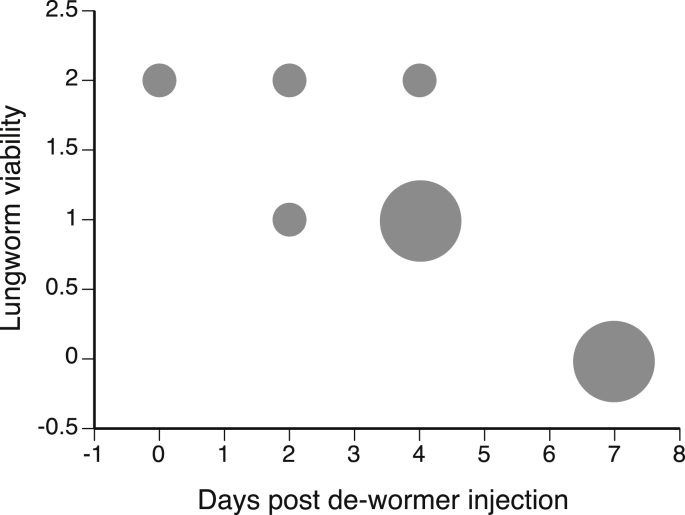
Effects of anthelmintic injection (Ivermectin) on the viability of adult lungworms (*Rhabdias pseudosphaerocephala* inside the lungs of cane toads (n = 10) hosts over a 7-day period following anthelmintic injection. Lungworm viability scores (see text for definitions) decreased with time since injection. The size of the symbols represents sample size of each score. Two toads had not been treated with anthelmintic when euthanized and were given scores of 0 days post injection.

Worm viability scores decreased with time since Ivermectin injection (n = 8, Spearman r = −0.78, p = 0.02; Fig. 2). Deterioration of worms was evident within 48 h of Ivermectin administration and increased up to the end of the experiment (7 days).

#### 3.2.2 Lung pathology and infection intensity

**Fig. 3:**
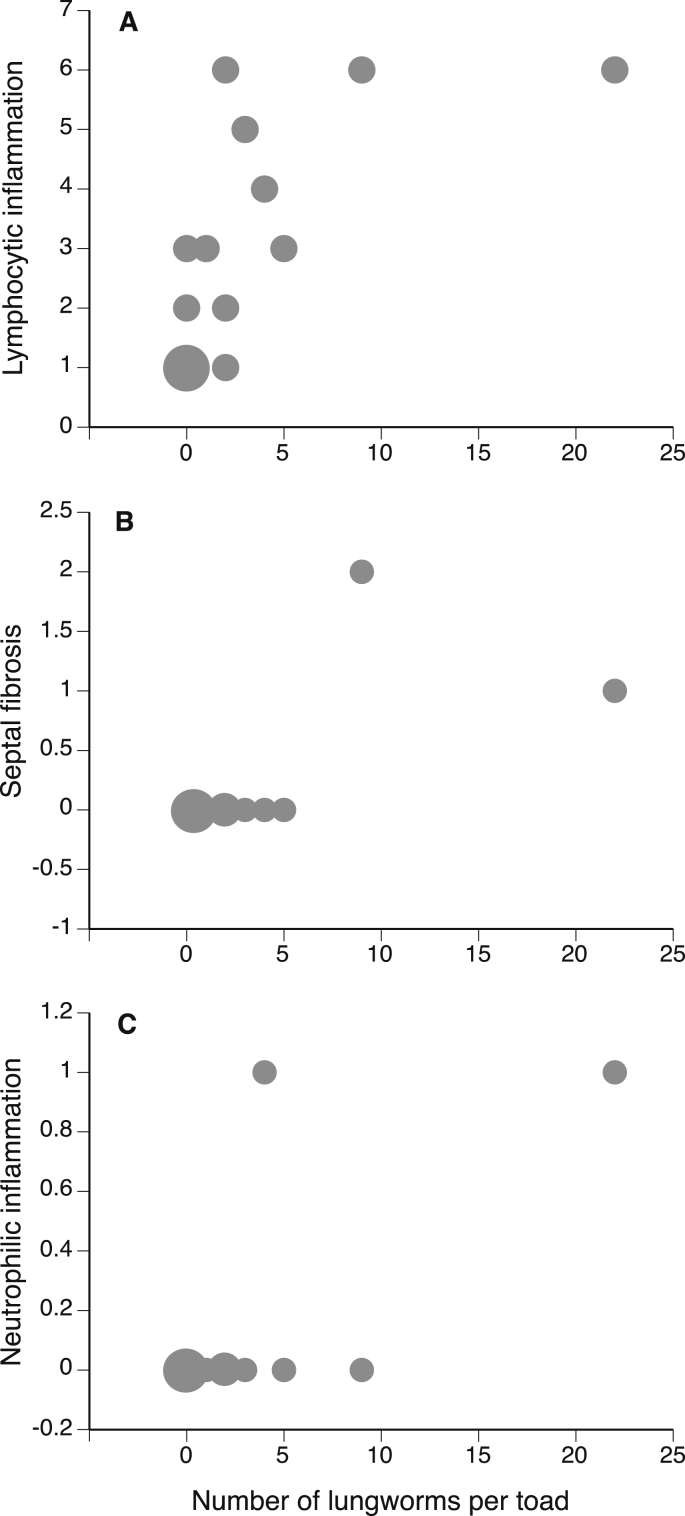
The effects of lungworm (*Rhabdias pseudosphaerocephala* infection level on lung pathology of 15 cane toads. (A) number of areas of inflammation predominated by lymphocytes, (B) number of areas of regional septal fibrosis, and (C) areas of inflammation predominated by neutrophils and macrophages. The size of the symbols represents sample size of each count.

*Rhabdias* infection induced subtle but significant pathological signs. Areas of inflammation associated with lymphocytes and neutrophils both increased with heavier *Rhabdias* infections (non-infected toads had intensity = 0: n = 15, Spearman r = 0.75, p < 0.01; Fig. 3A) and were associated with more areas of regional septal fibrosis (n = 15, Spearman r = 0.61, p = 0.02; Fig. 3B). Although only two melanomacrophages were observed, they both occurred in toads with heavy parasite infections, generating a barely significant correlation with infection level (n = 15, Spearman r = 0.52, p = 0.04; Fig. 3C).

### 3.3 Long-term effects of de-worming on immune response

**Fig. 4:**
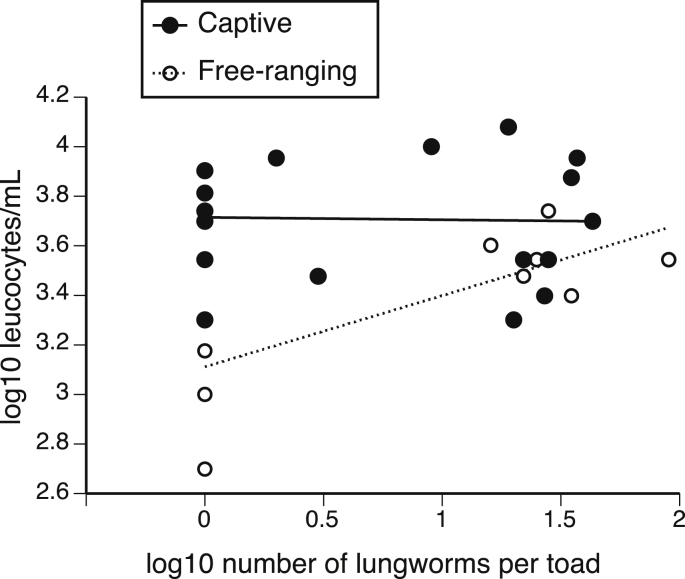
The effects of lungworm (*Rhabdias pseudosphaerocephala*) abundance on leukocyte concentrations in cane toads. Captive toads (n = 19) exhibited high leukocyte concentrations that were independent of the level of *Rhabdias* infection. In contrast, leukocyte concentrations in free-ranging toads (n = 11) increased with *Rhabdias* infection level.

The five free-ranging and 11 captive toads treated with anthelmintic drugs had significantly fewer *Rhabdias* than the six free-ranging and eight captive control toads (1.28 vs. 27.45; F_3,29_ = 84.53, p < 0.01). We assessed the effects of this resultant variation in infection intensity on immune measures. Concentrations of leukocytes responded to parasite infection intensity differently in captive vs. free-ranging toads (Table 2, Fig. 4). Captive toads exhibited high leukocyte concentrations that were independent of the level of *Rhabdias* infection. In contrast, leukocyte concentrations were low in non-infected free-ranging toads, but increased with *Rhabdias* infection level (Fig. 4).

**Table 2:** Effects of lungworm infection intensity (number of lungworms at dissection) and source (captive vs. free-ranging) on concentrations of blood cells and phagocytic and bactericidal ability of blood in adult cane toads (*Rhinella marina*). Significant values (p < 0.05) are shown in bold.

Trait	Effect	F_1,26_	p
**Blood cell concentration (per mL)**			
Erythrocytes	Free-ranging/captive	0.05	0.83
	ln-lungworm count	0.19	0.67
	Free-ranging/captive × ln-lungworm count	0.44	0.51
ln-leukocytes	Free-ranging/captive	19.3	<**0.01**
	ln-lungworm count	5.37	**0.03**
	Free-ranging/captive × ln-lungworm count	6.14	**0.02**
ln-neutrophils	Free-ranging/captive	3.09	0.09
	ln-lungworm count	3.32	0.08
	Free-ranging/captive × ln-lungworm count	0.87	0.36
ln-lymphocytes	Free-ranging/captive	49.64	<**0.01**
	ln-lungworm count	2.15	0.15
	Free-ranging/captive × ln-lungworm count	3.54	0.07
Neutrophil:lymphocyte ratio	Free-ranging/captive	14.9	<**0.01**
	ln-lungworm count	2.27	0.14
	Free-ranging/captive × ln-lungworm count	1.75	0.2
**Immune assay measure**			
Mean luminescence	Free-ranging/captive	21.22	<**0.01**
	ln-lungworm count	6.73	**0.02**
	Free-ranging/captive × ln-lungworm count	0.66	0.42
Maximum luminescence	Free-ranging/captive	19.29	<**0.01**
	ln-lungworm count	6.71	**0.02**
	Free-ranging/captive × ln-lungworm count	0.68	0.42
Time at maximum luminescence	Free-ranging/captive	1.68	0.21
	ln-lungworm count	6.13	**0.02**
	Free-ranging/captive × ln-lungworm count	3.55	0.07

Neutrophil numbers did not vary significantly with toad status or with infection level (Table 2). Lymphocyte concentration was higher in captive toads than in free-ranging toads but was not significantly affected by parasite infection level (Table 2). These contrasting patterns in neutrophil (N) and lymphocyte (L) concentration resulted in a significantly lower N:L ratio in captive toads than in free-ranging ones (Table 2).

**Fig. 5:**
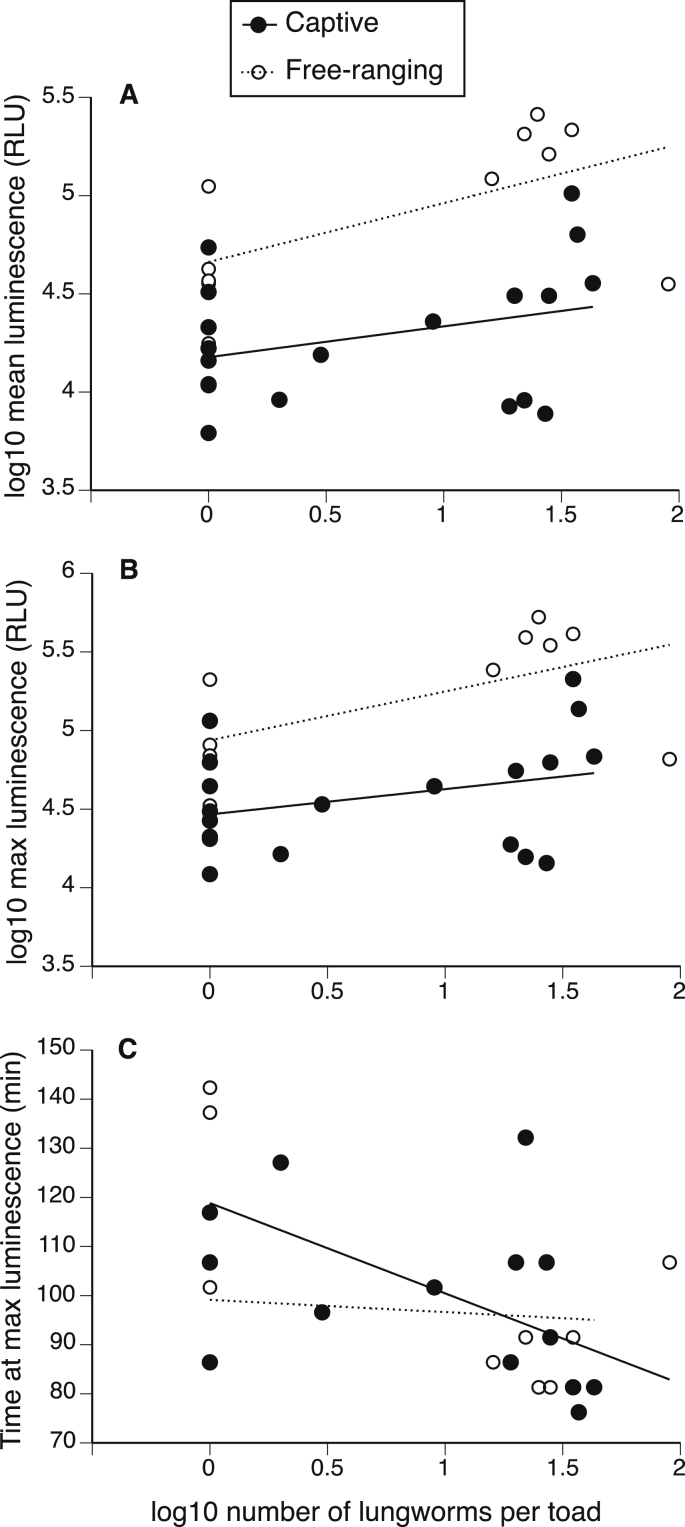
The effects of lungworm (*Rhabdias pseudosphaerocephala*) abundance on the phagocytic ability of the blood of 19 captive and 11 free-ranging cane toads. (A) mean luminescence over 160 min, (B) maximum luminescence over 160 min, and (C) time of maximum luminescence. RLU = relative luminescence units.

The whole blood of free-ranging toads had higher mean and maximum phagocytosis capacity than did the blood of captive toads (Table 2, Fig. 5A and B). Similarly, the blood of toads with heavier *Rhabdias* infections had higher phagocytotic ability, and reached peak levels sooner (Table 2, Fig. 5C).

Bactericidal capacity of plasma was not significantly related to parasite infection level, but free-ranging toads had marginally higher scores (p = 0.051) than did captive toads (Table 2).

There was no evidence that *Rhabdias* infection caused histological changes in the colon. The number of parasites infecting toads was unrelated to levels of lymphoplasmacytic (n = 11, Spearman r = 0.31, p = 0.35) or granulocytic (n = 11, Spearman r = 0.11, p = 0.74) infiltration of their colon mucosa.

### 3.4 Liver mass

**Fig. 6:**
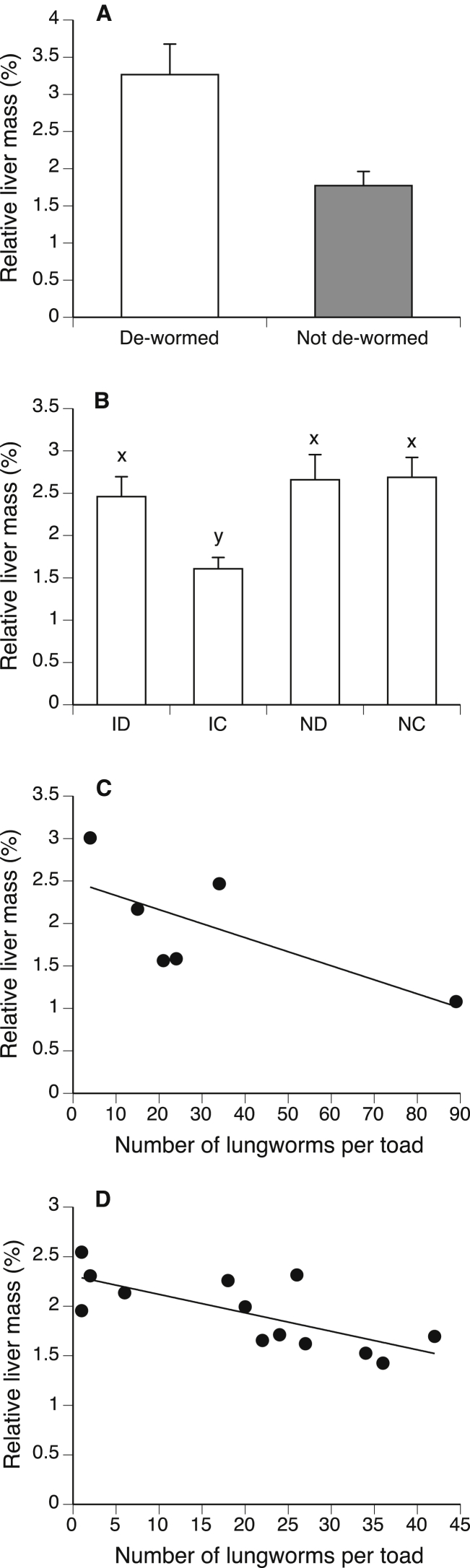
(A) The effects of anthelmintic treatment on relative liver mass of 5 de-wormed (open bars) vs. 6 not de-wormed (grey bars) free-ranging toads. (B) Effect of experimental treatment on relative liver mass of 49 captive cane toads. ID = infected, de-wormed (n = 11), IC = infected, control (n = 13), ND = non-infected, de-wormed (n = 13), NC = non-infected, control (n = 12). (C) Effect of *Rhabdias* infection intensity on relative liver mass of 6 free-ranging non-de-wormed toads. (D) Effect of *Rhabdias* infection intensity on relative liver mass of 13 infected captive cane toads given a control dose of amphibian ringers (IC). Bars show mean values ± 1 SE. Bars with the same alphabetical superscript are not significantly different from one another (p > 0.05).

Among the 11 free-ranging toads, de-wormed individuals had significantly larger relative liver masses than did toads that were not de-wormed (F_1,10_ = 64.28, p < 0.01; Fig. 6A). Similarly, among captive toads the group of infected individuals that were not de-wormed (IC) had significantly smaller livers than toads in the other three treatment groups (F_1,18_ = 66.10, p < 0.01; Fig. 6B). Among infected hosts, higher rates of infection were associated with a reduction in liver mass both in the free-ranging toads (F_1,5_ = 9.96, p < 0.01; Fig. 6C) and in captive toads (F_1,12_ = 11.21, p < 0.01; Fig. 6D).

## 4 Discussion

Previous studies on the effects of *Rhabdias* on cane toads have not reported any effects of the parasite on the host's immune configuration or response (Graham et al., 2012, Brown et al., 2016). However, those studies relied on correlational data from naturally infected individuals, or experimental infection protocols. Our study, utilizing experimental de-worming, detected significant effects of parasite infection level on host immune responses. These contrasting results suggest (i) that a host's immune configuration correlates with the level of infection, and may indicate either an influence on or a response to infection, and (ii) that the toads' physiology is perturbed by the chronic presence of adult *Rhabdias* in the lungs, not simply by larval migration through body tissues soon after the larva enters the host's body (as has been inferred by earlier studies: Kelehear et al., 2009, Pizzatto et al., 2010).

Over both the short-term and the long-term, anthelmintic treatment generated variation in lungworm infection intensity in both captive and free-ranging hosts, and induced multiple physiological and pathological changes. Some of these effects can be attributed to the removal of parasites, whereas others may relate to captivity and the injection of Ivermectin *per se* (rather than removal of parasites). For example, most immune parameters of the host were not affected over the short-term (<1 week) after de-worming, but maximum level of phagocytosis increased with time since Ivermectin injection. This increase was seen in non-infected as well as infected toads, suggesting that the effects of captivity and/or the drug itself (rather than the drug's effect on the target organism) triggered an up-regulation of the toad's phagocytic capability. Over the long-term, captive toads (regardless of infection intensity) exhibited an increase in leucocytes, lymphocytes and thus lower neutrophil:lymphocyte ratios than were seen in free-ranging toads, again, a potential response to captivity-induced stress. Thus, care must be taken in teasing apart the effects of parasitism on a host from the direct effects of experimental manipulation.

In the short-term (<1 week after anthelmintic injection), de-worming drugs had only minor immunological consequences on the host; the only significant immunological change caused by the de-worming of infected hosts was a shift in the bacterial killing ability (BKA) of host whole blood over time. Given the gradual decomposition of lungworm bodies inside the lungs of infected toads following de-worming, an increase in BKA may protect against bacteria-induced pneumonia. In addition, the decomposition of dead lungworms may release antigenic compounds into the blood stream and act as a trigger to up-regulate immune responses. The decrease in bactericidal ability seen in uninfected toads may be a compensatory response to the increase in phagocytic ability following anthelmintic injection. In the absence of a counter-acting stimulus (like dead worms), a trade-off between phagocytic and bactericidal capacities may occur (Brown and Shine, 2014, Brown et al., 2015a).

Pathological effects of infection on the cane toad host appeared relatively minor. Histopathological differences in lung tissue were detected between treatment groups, but the cellular changes were subtle. Similarly mild pathological consequences of infection were recorded in a South American population of cane toads parasitized by *Rhabdias paraensis*, with infection also resulting in detectable but minor tissue damage and inflammation (Santos et al., 2016). In keeping with the inference that pathological consequences of infection by *Rhabdias* in bufonid hosts often may be minor, *R. pseudosphaerocephala* infection caused no inflammation of the colonic epithelium tissue of toads. Thus, a tendency for lungworm infection to induce increased fecal water content and defecation rate in their toad hosts (Finnerty, 2017) may represent a manipulation of host physiology (Schmid-Hempel, 2011) rather than a simple histological consequence of disturbance to the colonic epithelium. The subtle pathology elicited by *Rhabdias* in their toad hosts may testify to their long co-evolutionary history.

In contrast to these impacts over the short-term, longer-term (>2-month) monitoring of free-ranging hosts post injection revealed significant shifts in immunological components that differed between hosts of varying infection intensity. Leukocyte (white blood cell) concentrations increased with parasite burden and the concentration of phagocytic neutrophils, although statistically non-significant (p = 0.08), tended to be higher among toads with heavier infections. An increase in neutrophils may have elevated the phagocytic ability of infected whole blood. Although we do not know whether or not increased phagocytic capacity of toad blood is effective at fighting *Rhabdias* infection (given that adult lungworms are too large to be directly phagocytozed), this increase could induce mild inflammation in the lungs of infected toads. Regardless, this long-term up-regulation of immunological components (and its associated energetic costs) may contribute to the ecological and behavioral consequences of infection recorded in our concurrent studies (Finnerty, 2017, Finnerty et al., 2017).

Over the long-term, continued infection also caused a reduction in liver mass (a major energy storage organ: Smith, 1950, Morton, 1981, Scapin and Di Giuseppe, 1994, Isehunwa et al., 2016, Silvester et al., 2017) in both captive and free-ranging toads, with the decrement in liver mass proportional to infection intensity. This reduction in liver mass may reflect the energetic costs of an up-regulation of immune components combined with reduced movement and thus prey consumption (Finnerty et al., 2017). Unfavorable conditions during the abnormally dry season when our study was conducted (low food, low moisture, high toad density) may have imposed further stress and energetic constraints on toads, and thus amplified any negative effects of parasitism. Such reduced energy intake, immune-system up-regulation associated with increased parasitic infection intensity, and unfavorable (arid) conditions may have contributed to the substantial negative impact of lungworms on host growth and performance (Finnerty et al., 2017).

In order to interpret the impacts of parasite infection on the biology (including, ecology and behavior) of their hosts, it is vital to understand the proximate consequences of infection. Experimental de-worming offers a powerful means to quantify such impacts. Specifically, manipulative de-worming generates experimental variation in infection intensity, which overcomes the inherent problems associated with studying naturally occurring variation in infection. Understanding the histopathological and immunological impacts of infection can clarify the proximate consequences of parasitism, and facilitate a more holistic understanding of parasite–host interactions.
